# Golgi Complex form and Function: A Potential Hub Role Also in Skeletal Muscle Pathologies?

**DOI:** 10.3390/ijms232314989

**Published:** 2022-11-30

**Authors:** Luana Toniolo, Giuseppe Sirago, Nicola Fiotti, Emiliana Giacomello

**Affiliations:** 1 Laboratory of Muscle Biophysics, Department of Biomedical Sciences, University of Padova, 35131 Padova, Italy; 2 Department of Medicine, Surgery and Health Sciences, University of Trieste, 34149 Trieste, Italy

**Keywords:** muscular dystrophy, Limb girdle muscular dystrophy, dystrophin associated protein complex, early secretory pathway, glycosylation, Golgi Complex

## Abstract

A growing number of disorders has been associated with mutations in the components of the vesicular transport machinery. The early secretory pathway consists of Endoplasmic Reticulum, numerous vesicles, and the Golgi Complex (GC), which work together to modify and package proteins to deliver them to their destination. The GC is a hub organelle, crucial for organization of the other secretory pathway components. As a consequence, GC’s form and function are key players in the pathogenesis of several disorders. Skeletal muscle (SKM) damage can be caused by defective protein modifications and traffic, as observed in some Limb girdle muscular dystrophies. Interestingly, in turn, muscle damage in Duchenne dystrophic SKM cells also includes the alteration of GC morphology. Based on the correlation between GC’s form and function described in non-muscle diseases, we suggest a key role for this hub organelle also in the onset and progression of some SKM disorders. An altered GC could affect the secretory pathway via primary (e.g., mutation of a glycosylation enzyme), or secondary mechanisms (e.g., GC mis-localization in Duchenne muscles), which converge in SKM cell failure. This evidence induces considering the secretory pathway as a potential therapeutic target in the treatment of muscular dystrophies.

## 1. Introduction

In recent years, study of the correlation between the secretory pathway and inherited human diseases has become increasingly important, allowing for a link to a growing number of disorders and mutations in the components of the secretory pathway [1,2,3].

In the eucaryotic cell, the secretory pathway is constituted by several components with the fundamental tasks to receive, modify, and package proteins and lipids to drive them to their final destinations. In the conventional secretion pathway, proteins are synthetized in the rough endoplasmic reticulum (ER), they leave the ER at the level of the ER- exit sites (ERES) and are transported in COPII vesicles to the Golgi Complex (GC), where they undergo post-translational modifications (e.g., glycosylation) and are packed into post-GC vesicles to reach their target. Defective proteins return to the ER via COPI vesicles. The early secretory pathway, which consists of the ER, ERES, COPII and COPI vesicles, and the GC is the first secretory compartment that the proteins transit to reach their final destination. Since this part of the secretory pathway fulfill the task of selectively transporting native proteins to reach their final target, the ER to GC interface is a fundamental checkpoint for protein scrutinization [4].

In mature skeletal muscle (SKM) cells, the architectural disposition of the early secretory components is unique: the GC is formed by very small elements, localized near the nucleus and in the fiber in close association with ERES in a fiber-type dependent fashion [5,6,7]. This arrangement is the result of a reorganization process occurring during SKM differentiation, which leads to the formation of small GC elements localized near the ERES [5,6]. How this process takes place is not completely clear; however, our recent work [5], in agreement with data on non-muscle cells [8], suggests that during the SKM differentiation process, the GC plays a central role in the positioning and regulation of ERES and other early secretory pathway components. 

Actually, these data agree with the evidence that, in other cell types, the GC is not only the central organelle for glycosylation, but it exerts a hub function in the regulation of the transport via the modulation of the assembly and localization of vesicles, as well as ERES distribution [9]. This provides for the GC a key role in the onset and progression of several diseases [10,11] and calls for the investigation of the correlation between SKM diseases and the form and function of the GC.

## 2. The Early Secretory Pathway and Skeletal Muscle Dystrophies

Congenital muscular dystrophies are a heterogeneous group of inherited disorders characterized by progressive muscle weakness and wasting [12,13]. Although a large number of mutations to the distinct components of the dystrophin associated protein complex (DAPC) have been shown as causative for several forms of dystrophy [12], there is growing evidence that defects in the biosynthetic pathway, such as protein modification, folding or intracellular traffic, have an important influence on SKM integrity [1,2].

Interestingly, despite the numerous proteins involved in the early secretory pathway, the very low level of mutations associated with a disease indicates its essential role in the cell physiology. 

Accordingly, the literature reports several forms of muscular dystrophies and myopathies associated with mutations to genes involved in post-translational modifications (e.g., glycosylation enzymes [14,15,16]) in membrane traffic (e.g., Cav3 [17], TRAPPC11 [18,19]) and cell membrane remodeling (e.g., dysferlin [20]), while there are few examples of mutations to GC resident proteins or proteins involved in the ER to GC transport. Few exceptions are the description of the mutation to the GC matrix protein 130 (GM130) [21,22] and to the vesicle fusion protein BET1 [23].

In general, due to the ubiquitous expression of most of these proteins, the damage to the SKM tissue can be one of the features in a very complex clinical condition, as largely described for some alpha-dystroglycanopathies [13,14,15] and recently reported in patients with mutations to the GC matrix protein GM130 [21,22] and the vesicle fusion protein BET1 [23].

## 3. The Golgi Complex: A Hub Organelle in the Early Secretory Pathway

The GC is involved in protein quality control and is the fundamental organelle for the glycosylation of numerous proteins. To this aim, the GC is a highly dynamic organelle, organized in flattened polarized cisternae, organized in a *cis* region facing the ER and *trans* region directed toward the plasma membrane [8,24,25]. Transport between the ER and the GC is regulated by the COP II and COP I coat vesicles. The conventional protein secretion pathway entails the translocation of proteins directly from the ER or via COPII vesicles to the *cis* face of the GC (anterograde traffic), where they are modified and released via the *trans* face, to be then directed to the plasma membrane. Defective proteins are transported in COPI vesicles in the retrograde traffic pathway [26].

There is convincing evidence that the maintenance of the GC structure is important for its own function and that several diseases are correlated with modifications of the GC morphology and localization [10,11,25]. In fact, an alteration of its morphology potentially leads to damages in the organization of the sub-compartments and can introduce an impairment of the post-translation processing as a consequence. In turn, the accumulation of improperly modified proteins affects GC organization, vesicles assembly, and protein traffic itself [24,27]. Altogether, this evidence confers a central role to the GC [9].

A comparable role for the GC could be also advanced in the context of the SKM cell because, as suggested by Kanagawa in a description of alpha-dystroglycanopathies [14], proteins that also play a role in the structural maintenance and function of the GC may result in alterations to the glycosylation machinery, affecting the biosynthetic pathway of dystroglycans. 

In support to the important role of the GC in the SKM cell, our recent work showed that in in vitro differentiating myoblasts, the depletion of GM130 interferes with GC morphology and ERES organization. It strongly inhibits the transport of M-cadherin, a membrane protein involved in myoblast fusion [28], to the plasma membrane, which remains confined at the ER level. As a final effect, the loss of M-cadherin at the plasma membranes significantly decreases the efficiency of cell fusion [5]. This suggests that the re-organization of ERES, modulated by the GC matrix protein GM130, plays a crucial role in the efficient transport from ER to GC of differentiation-specific cargos during SKM differentiation and provides the first example of specific traffic inhibition from the ER to GC in SKM cells.

Interestingly, it has been reported that mutations to the GC matrix protein GM130, in a pediatric patient from consanguineous parents, induce developmental delay, neuromuscular disorders and muscular damage [21]. Work from Shamseldin and collaborators showed that the mutation in the gene sequence produces a GM130 truncated protein, which is either not produced or very unstable, resembling a null mutation where GM130 functions are likely lost [21]. 

Although GM130 is a ubiquitous protein, and therefore the clinical picture is complicated with the malfunctioning of multiple organs and system (e.g., the central nervous system) [13,22,29], in vitro experiments, together with the characterization of the human mutated protein, suggest that GM130 plays an important role in SKM functioning. Moreover, these data suggest that the strict coordination among the distinct components of the early secretory pathway (e.g., ER to GC transport) is essential for a correct muscle physiology. 

**Figure 1:**
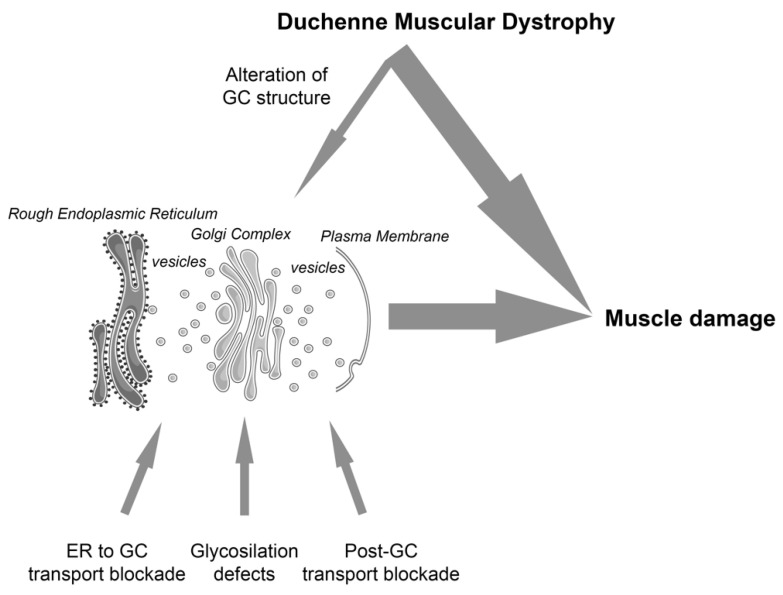
The GC is a central organelle in the biosynthetic pathway. We suggest that GC integrity and functionality contributes to the pathological mechanisms of muscular dystrophies via direct (bottom arrows highlight three different conditions that affect protein traffic in the context of muscular dystrophies: ER to GC blockade [5,21,22]; glycosylation defects [14,16]; post GC transport blockade [18,19]) or secondary mechanisms (top arrow; [30,31,32]), contributing to muscle damage. The figure was partly generated using Servier Medical Art, provided by Servier, licensed under a Creative Commons Attribution 3.0 unported license.

On the other hand, it has been shown that the alteration of muscle specific proteins, not directly involved in the early secretory pathway, could also have an impact on GC form and function. This is the case of the Duchenne muscular dystrophy (DMD), where the loss of dystrophin is accompanied by the alteration of the morphology and mis-localization of the GC, [30,31], with a parallel modification of the microtubule network [32]. Based on data showing the strict relation between form and function in non-muscle cells [10,11,25,27,33], we hypothesize that the alteration of GC morphology in DMD muscle cells introduces a secondary effect that, in a chain-reaction mode, may amplify the progression of the pathology (Figure 1).

In conclusion, in light of the above-mentioned correlation between GC form and function in non-muscle cells [10,11,27,33], according to our observations showing the role of GC form and function during muscle differentiation [5] and the loss of an adequate GC morphology in dystrophic muscle fibers [31,32], we suggest that the GC integrity and functionality can potentially contribute to the pathological mechanisms of muscular dystrophies in a dual way (Figure 1). Either it may contribute directly (e.g., aberrant glycosylation due to a mutation of a GC resident enzyme, such as in alpha-dystroglycanopathies), or/and can have a secondary role (e.g., GC mis-localization in Duchenne dystrophy). Both conditions potentially lead to an altered early secretory pathway. However, in the latter situation, the inefficiency of the protein traffic is an addition to the preexisting pathological mechanisms, which could lead to an amplification of the pathological response, possibly contributing to a worse progression of the SKM disorder.

## 4. The Components of the Early Secretory Pathway as Therapeutic Targets

Either for direct or secondary contributions, the resulting effect of a non-working early secretory pathway is an accumulation of proteins [4]. 

Similar to an airways traffic jam, where the passengers take alternative flights to reach their final destination, we can try to manipulate some of the components of the intracellular traffic by exploiting alternative routes and/or hasten the traffic, allowing proteins to reach their destination. 

Potentially, different approaches can be applied to reduce the consequences of a defective secretory pathway [34]. These strategies could be summarized in the following categories: (1) agents that modulate the proteostasis, able to interact with the protein quality control, or to reduce ER and cellular stress; (2) a genetic approach to modify mutated proteins [31]; (3) exploitation of the unconventional protein secretion route [35]. 

Some compounds have already been identified (e.g., in the treatment of Cystic Fibrosis [34]); however, the application of drugs that target the secretory pathway requires further study, and, therefore, this therapeutic approach is still poorly pursued.

It can be suggested that the application of secretory pathway regulators could be of help in also treating muscular dystrophies. Evidently, the choice of the treatment depends on the type of disease and whether the secretory pathway defects result from a direct (e.g., Fukuyama myopathy) or secondary effects (e.g., Duchenne muscular dystrophy). Actually, in the latter situation, the therapeutic targeting of the secretory pathway is not sufficient to prevent or delete the principal effects of a defective protein (e.g., dystrophin), but could have an adjuvant effect by modulating the load of the above-mentioned secondary effects (Figure 1).

However, since the different components of the secretory pathway are highly connected, it is not excluded that a single strategy can have multiple effects and can be helpful for several muscular pathologies.

## 5. Conclusions

In this perspective, we briefly reported and discussed data on the relevance of the early secretory pathway and its hub organelle, the GC, in the context of muscular dystrophies. Despite the paucity of data on the secretory pathway in the SKM cell, observations from the literature lead us to suggest that the integrity of the GC and vesicular components is necessary for maintenance of the form and function of this tissue. 

Based on observations in other cell types, where the alteration of the secretory pathway function results in unproper targeting and the accumulation of proteins, we hypothesize that the secretory pathway can be a potential therapeutic target in the treatment of SKM diseases. 

A desirable outcome of future research is to gain further understanding of mechanisms regulating the secretory machinery in the SKM and to test the possibility of targeting the secretory pathway to ameliorate SKM function in pathologic conditions. 

## Data Availability

Not applicable.
